# Pre-hospital stroke monitoring past, present, and future: a perspective

**DOI:** 10.3389/fneur.2024.1341170

**Published:** 2024-03-22

**Authors:** Hilla Ben Pazi, Shady Jahashan, Sagi Har Nof, Samuel Zibman, Ornit Yanai-Kohelet, Limor Prigan, Nathan Intrator, Natan M. Bornstein, Marc Ribo

**Affiliations:** ^1^Avertto Medical Ltd., Aderet, Israel; ^2^Neurosurgery, Galilee Medical Center, Nahariya, Israel; ^3^Neurosurgery, Rabin Medical Center, Petach Tikva, Israel; ^4^Neurosteer Inc., New York, NY, United States; ^5^Stroke Unit, Neurology, Shaare Zedek Medical Center, Jerusalem, Israel; ^6^Tel Aviv Medical School, Tel Aviv University, Tel Aviv, Israel; ^7^Stroke Unit, Neurology, Barcelona, Spain

**Keywords:** stroke, monitoring, pre hospital, early intervention, home care

## Abstract

Integrated brain-machine interface signifies a transformative advancement in neurological monitoring and intervention modalities for events such as stroke, the leading cause of disability. Historically, stroke management relied on clinical evaluation and imaging. While today’s stroke landscape integrates artificial intelligence for proactive clinical decision-making, mainly in imaging and stroke detection, it depends on clinical observation for early detection. Cardiovascular monitoring and detection systems, which have become standard throughout healthcare and wellness settings, provide a model for future cerebrovascular monitoring and detection. This commentary reviews the progression of continuous stroke monitoring, spotlighting contemporary innovations and prospective avenues, and emphasizes the influential roles of cutting-edge technologies in shaping stroke care.

## Introduction

With increasing longevity, preserving well-being is recognized as a global challenge (UNDP3 goal: reducing premature mortality from non-communicable diseases). Stroke is among the world’s leading causes of long-term disability and death, yet it is treatable if diagnosed within the ‘Golden Hour.’ Unfortunately, most patients do not reach medical facilities within that time window. To attain this global challenge goal, pre-hospital stroke monitoring is essential. This perspective delves into the emerging field of continuous stroke monitoring at our doorstep.

## Past

While treatment modalities for stroke have evolved from leeches in the medieval ages to advanced thrombectomy interventions, early detection remains behind, relying mainly on clinical observation. Emphasis has been on implementing methodologies for prompt stroke management in the hospital setting (1). Yet only 15–32% of patients presenting with treatable ischemic stroke arrive in time (2).

The answer to improved onset to treatment time (OTT) must include effective home monitoring for people at risk, as seen today in the cardiovascular field. Today, with technological advancements, monitoring has permeated the home and wellness arenas. Wearable devices like the Apple Watch allow continuous data flow and collection of cardiovascular parameters without significant inconvenience to the user. Insertable diagnostic devices provide a more invasive yet comprehensive way to monitor cardiac heart rhythm (3). Medical advancements have created improved sensor technology, which has increased the data generated. Artificial intelligence (AI) today plays a pivotal role in analyzing this data. Cloud-based telemonitoring with cybersecurity advancements has fostered trust in cloud solutions, allowing patients and doctors remote yet real-time access to health data, leading to monitoring, detection, and alerts.

**Figure 1:**
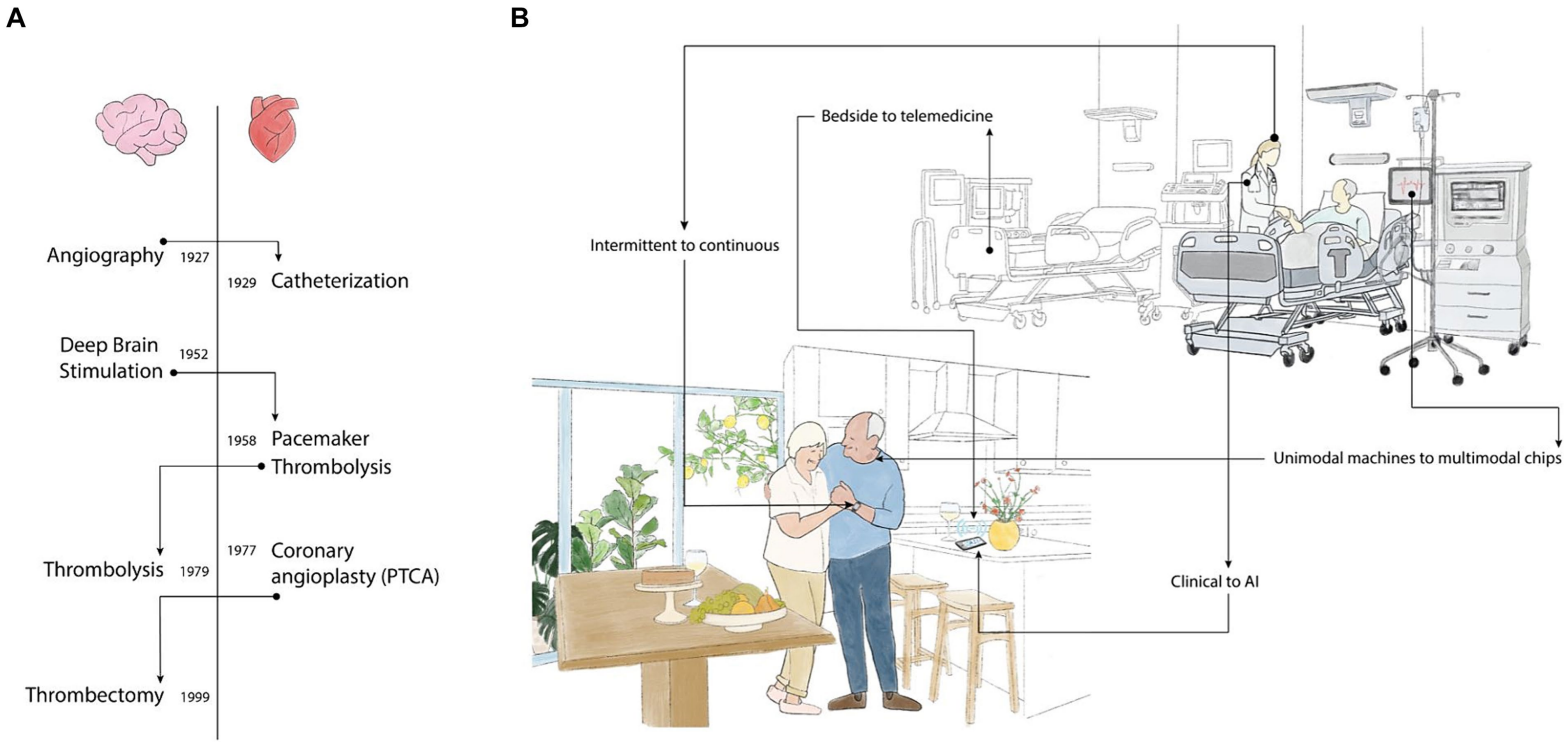
**(A)** Shifting technology: Flow of technologies from cardiac to neurology and vice versa over the years. Adaptations were faster in earlier years and in the Cardiovascular field. **(B)** Trends in Stroke Monitoring-shifting from nonspecific referral to LVO detection. Conventional Healthcare is introducing new trends, shifting from intermittent human detection to continuous real-time monitoring, hospital bedside to home telemedicine monitoring, relying less on clinical signs and more on AI-based algorithms. Unimodal machines to small multimedia chips; health alerts to wellness activities.

Throughout medical history, innovative ideas in the cardiovascular field have been adopted with time in the cerebrovascular arena and vice versa (Figure 1A). Considering the advancements in cardiovascular monitoring, we expect cerebrovascular guidelines (4) to shift from secondary prevention to dedicated stroke monitoring devices according to evolving medical trends (Figure 1B).

## Present

In managing inpatients at high risk for stroke, current practices focus on risk assessment, identifying and addressing underlying causes in various clinical scenarios. These scenarios include patients in stroke units recovering from cerebrovascular attacks, individuals experiencing vasospasm due to aneurysmal subarachnoid hemorrhage, and those with reversible cerebral vasoconstriction syndrome (RCVS). Additionally, it encompasses patients undergoing high-risk procedures or surgeries or those admitted to the Intensive Care Unit (ICU). Regularly monitoring these patients involves recurrent assessments of neurological status and continuous monitoring of vital signs. Management strategies include blood pressure control, antiplatelet medication, and early mobilization as key preventive measures against in-hospital stroke. Imaging is recommended to assess evolving cerebrovascular events when there is a noticeable change in a patient’s neurological status (in awake patients) or significant alterations in vital signs (in sedated patients). However, factors like sleep, sedation, and ICU-related psychosis limit clinical assessments, posing challenges to the early detection and management of stroke in these high-risk patients.

## The need

Large vessel occlusions (LVOs) occur when a blockage is created in one of the major arteries supplying blood to the brain, resulting in a high risk of severe disability or mortality. Stroke due to a large vessel occlusion is treatable, and treatment is associated with remarkably improved outcomes. However, eligibility for treatment and the likelihood of good outcomes among those treated are highly time-sensitive; any delay in intervention correlates with increasingly poorer outcomes (5).

In the context of emergency stroke care, the prioritization of sensitivity over specificity is a strategic decision. The medical community in neurology is inclined to accept a higher rate of false positives to substantially reduce the risk of false negatives, especially for identifying LVOs. Moreover, since the significant physiological changes caused by LVOs are relatively easy to detect, most monitoring devices designate LVOs as their primary monitoring target.

## Clinical blind spots

There are certain situations where an LVO will not be immediately detected, particularly during sedation, sleep, and single living, in which case the diagnosis will be delayed.

### Sedation

The risk of stroke is exceptionally high around invasive procedures, highest in cardiovascular procedures (over 10% in some procedures) (6), and varies in other types of surgeries (0.05–4.4%) (7). Approximately 4 to 17% of all stroke cases occur during hospitalization. Morbidity and mortality are higher in the peri-operative arena.

### Sleep

‘Wake-up’ strokes occur during sleep and only present upon awakening when observable clinical changes can be detected, thus delaying diagnosis. These account for about 20% of all strokes (8). This happens even in hospital patients admitted for a cerebrovascular event such as a transient ischemic attack (TIA), which has a subsequent 5% risk of recurrent stroke within the first 48 h.

### Living alone

People over 60 tend to live alone, reaching 28% in the United States and Europe (9). Their numbers are rising with longevity and social changes. Stroke risk increases with age. Many people who live alone cannot recognize or act if they are affected by a stroke. Responsive monitoring, detection, and alerts lower the chance of such situations and lower the personal stress that accompanies these events.

## The opportunity

### Vital signs monitoring

Currently, no monitoring device in routine use can detect strokes (Table 1). Hospitalized patients are typically monitored using vital signs devices that track parameters such as ECG, blood pressure, and oxygen saturation. These devices provide cardiovascular alerts but are not designed to detect strokes. While changes in vital signs may occur during a stroke (10, 11), these may be subtle and nonspecific.

**Table 1:** Current stroke monitoring technologies.

Technology	Trans-Cranial-Doppler	EEG	SEPs	Accelerometer	NIRS	Piezoelectric
Passive/Active	Active	Passive	Active	Passive	Active	Passive
Type	Ultrasound device	Wearable	Wearable	Wearable	Bedside	Monitor/ Insertable
Preventive/reactive	Reactive	Partly	Partly	Reactive	Reactive	Preventive
Real-time Alert before neurological damage	Early/ischemia	Early/ischemia	Early	Late/clinical signs	Late/bleeding	Before clinical signs
Arena	Hospital	Home/hospital/ambulance	Hospital	Home	Hospital/ambulance	Home/hospital/ambulance
Continuous monitoring	Once suspected	Yes	Once suspected	Yes	Once suspected	Yes/No
Registration/indication	FDA: cerebral blood flow	Clinical trials	Clinical trials	CE FDA: Telemonitoring	FDA/hematoma	Clinical trials
Location	Head	Head	Head	Wrists	Head	Neck/Head
Portable	No	Bedside/Y/N	Yes	Yes	No	Yes

There are intentions to use vital signs AI analysis to detect and alert for stroke. Several wearable vital signs companies provide remote monitoring solutions for cardiac and other health conditions. While their primary focus is heart-rate monitoring, these companies are interested in neurological monitoring and exploit their expertise in remote monitoring and AI knowledge to enhance stroke detection. They have been proven effective in long-term insertable monitoring (12).

### EEG

EEG is a promising method. It is small and inexpensive, with sensors that can capture various signals. Its continuous nature makes it a promising candidate for stroke monitoring. Brain monitoring for anesthesia depth has been used since 1986, known as the Bispectral Index (BIS; 4 channels). Low BIS values with low blood pressure were associated with peri-operative stroke in cardiothoracic surgery (11); thus, BIS mitigates perfusional stroke, a small subset of periprocedural stroke. Prospective studies are warranted to check if BIS can reduce the incidence of peri-operative stroke.

Currently, EEG has not shown success in differentiating between ischemic and hemorrhagic stroke, even though it shows some differences in the distribution of brain activity, where the reduction in activity in the case of ischemic stroke is more local and is more diffuse in the case of hemorrhagic (13). New advanced technologies and various AI tools detecting LVOs in ambulances would allow direct referral of patients with thrombectomy abilities to stroke centers. Clinical studies with an EEG cap (8 electrodes) to detect LVOs in the ambulance setting found that frequency and Brain Symmetry Index (0 = perfect symmetry between the two hemispheres; 1 = complete asymmetry) provided the best accuracy (*n* = 311, accuracy 91%) (14).

A lightweight EEG 4-channel headband paired with a smartphone app to detect stroke changes during sleep at home has been registered (ClinicalTrials.gov 05669456). Somatosensory evoked potential (SEP) detects EEG changes in the stroke arena that may be related to the integrity of the somatosensory pathway and the extent of brain injury. These changes appear following revascularization in acute stroke and may assist in determining eligibility for thrombectomy (15). As SEPs require episodic stimulation, it will probably be effective as an add-on feature corroborating continuous EEG monitoring once suspicion is raised.

While not developed for stroke, an integrated brain-machine interface platform using deep learning can detect stroke-related changes better than noninvasive EEG methods and may enhance the previously described AI monitoring (16). It may also provide treatment. Future developments may determine if efficacy would be sufficient to justify a brain implant device.

### Optic sensors

Near-infrared-spectroscopy (NIRS) is a non-invasive imaging technique that measures changes in the brain’s cortical tissue’s oxygenated and deoxygenated hemoglobin concentration. Advances in fibreless, whole scalp diffuse optical tomography systems improved wearability (17) and can be used for natural/social behaviors (18). The existing clinical literature suggests NIRS plays a vital role in the real-time monitoring of motor function recovery and cerebral hemodynamic change, such as identifying a recanalization following mechanical thrombectomy. Current NIRS monitoring focuses on brain function and oxygenation. Given these abilities, NIRS is being investigated to assess cerebrovascular diseases, including stroke (19). A portable handheld optical device has been developed and registered for brain intracranial hemorrhage assessment in emergency settings (20). Future development may combine EEG monitoring for brain function assessment with NIRS for cerebral hemodynamics and stroke monitoring.

### Imaging: ultrasound doppler

Transcranial Doppler (TCD), which assesses blood flow velocities in the middle cerebral-artery (MCA), requires experienced personnel, readily available in some clinical settings. Several devices were developed to overcome the need for experienced bedside technicians; some developed an automated TCD, detecting MCA flow automatically, with detection abilities similar to registered technicians. Automated TCD results also match CT angiogram (*n* = 33, 83% sensitivity, 90% specificity) (21). The efficacy of an automated alert mode and broader uses in other sedated populations at risk remains to be determined.

### Motion-based sensors

Several offer continuous wearable devices to monitor hand movements. This method can accurately record changes in hand motion in people who suffered a stroke (22). One differentiating aspect of this approach is that some devices require time to establish a baseline while others detect the presence of asymmetry. The former may limit utility if the time for baseline acquisition is long, while the latter cannot be used in patients with baseline asymmetric arm strength (e.g., post-stroke). There is no evidence of real-time detection. The home monitoring concept requires that, once a change is detected, the person must follow instructions for a short examination to corroborate the alert. As some products are registered (FDA/CE) and marketed, it will shortly be determined if the detection tool yields efficient information early enough to impact clinical outcomes. Various multifaceted watches can monitor falls, which may be essential for a person experiencing an acute stroke. Falls, however, are relatively late clinical consequences of stroke and often result in the inability to contact emergency services.

### Head and neck pulse monitoring

LVOs to the brain generate measurable flow changes. This offers a unique value - the ability to detect changes indicating stroke risk before neurological damage occurs. There are several ways to detect flow change.

One method is a cranial accelerogram based on ballistocardiography, a measurement of the forces of the cardiac cycle. Clinical studies demonstrated a correlation between an AI-based algorithm analyzing a combination of fiduciary points of the head pulse movement and the existence of an LVO according to imaging results (*n* = 92; 78% sensitivity, 99% specificity) (23) and is tested for pre-hospital settings for triaging people with LVOs to centers with thrombectomy abilities.

Another method is detecting carotid pulse wave changes in stroke (24, 25). Clinical studies are performed to check the ability of continuous carotid artery monitoring devices to detect LVOs (personal communication). An AI-based algorithm detects waveform changes that indicate increased carotid stiffness. The detection of an obstruction before neurological injury has the potential to alert stroke and enable early intervention within the golden hour.

### Future

The inefficiencies of current stroke detection methods highlight the need for innovative approaches. All current stroke monitors (Table 1) cover a specific angle, such as LVO, and use one modality. As with cardiovascular vital signs monitoring, we expect an integration of various parameters and modalities, including NIRS, EEG, motion-based sensors, and carotid artery monitoring, to enable a holistic picture of cerebral health. Integrated data from these devices can provide early, precise, and actionable insights for better stroke prediction and intervention.

As sensor technology advances, we can anticipate creating compact, user-friendly, and minimally invasive devices, facilitating comfortable 24/7 monitoring and reducing the barriers to people adopting these devices. With the proliferation of 5G and subsequent generations of internet connectivity, real-time remote IoT (Internet of Things) monitoring will become seamless, bridging the gap between patients and clinicians and ensuring immediate intervention with telemedicine beginning even before a patient reaches a healthcare facility. Patients and providers must be educated on their usage, interpretation, and actionable steps, ensuring the realization of the full potential of these tools.

Integrating these devices with other health monitors and electronic health records can provide a comprehensive health overview, thus aiding in timely intervention for stroke and associated comorbidities. The focus is on creating economic devices accessible to a broader population, especially in low-and middle-income countries with significant stroke burdens.

The quintessential stroke monitor of the future warrants stringent criteria. Foremost, it should understand how to detect LVOs within the pivotal “golden hour” (26). It should be adept at securely transmitting data around the clock, whether the patient is at home or navigating public spaces. Its design should inspire regular compliance through wearability or the potential to insert these devices without compromising long-term safety. Furthermore, it should be multifaceted, capable of concurrently monitoring crucial cardiac events such as atrial fibrillation alongside strokes, with impeccable sensitivity and specificity. An invaluable addition would be GPS functionality, streamlining patient location and referral.

The development of a dedicated monitoring device has the potential to enhance stroke management by providing real-time monitoring and timely alerts, substantially impacting patient outcomes. Integrated brain-machine interface adds another layer as it attempts not only to detect but also to treat. While transcranial-magnetic stimulation is already used for stroke rehabilitation, other innovative interventions are expected to evolve. This paradigm shift promises to mitigate the morbidity, mortality, and long-term disability associated with strokes, heralding a brighter, more proactive future in stroke care.

## Author contributions

HB: Conceptualization, Funding acquisition, Writing – original draft. SJ: Writing – review & editing. SH: Writing – review & editing. SZ: Software, Writing – review & editing. OY-K: Conceptualization, Writing – review & editing. LP: Conceptualization, Writing – review & editing. NI: Writing – review & editing. NB: Supervision, Writing – review & editing. MR: Writing – review & editing.
